# Virologic insights from the phase 3 REDPINE trial: remdesivir in renally impaired and immunocompromised patients with COVID-19

**DOI:** 10.1128/aac.01912-25

**Published:** 2026-07-10

**Authors:** Lauren Rodriguez, Kristen Andreatta, Shuguang Chen, Yu Hu, Jiani Li, Ross Martin, Dong Han, Jasmine Moshiri, Jason K. Perry, Meghan E. Sise, Jose Ramon Santos, Markus Plate, Mark J. McPhail, Yiannis Koullias, Robert H. Hyland, Charlotte Hedskog

**Affiliations:** 1 Gilead Sciences, Inc. 2158 , Foster City, California, USA; 2 Division of Nephrology, Massachusetts General Hospital 2348, Boston, Massachusetts, USA; 3 Fight Infections Foundation, Service of Infectious Diseases, Hospital Universitari Germans Trias i Pujol 16514, Badalona, Spain; 4 Department of Medicine, Division of Infectious Diseases, Weill Cornell Medicine 639780, New York, New York, USA; 5 Institute of Liver Studies, King's College Hospital 111990, London, United Kingdom; Chinese Academy of Medical Sciences & Peking Union Medical College, Beijing, China

**Keywords:** COVID-19, remdesivir, SARS-CoV-2, viral shedding, virologic resistance

## Abstract

Remdesivir inhibits the SARS-CoV-2 RNA-dependent polymerase (Nsp12). Here, we present virology analyses from the Phase 3 REDPINE trial in participants with impaired kidney function hospitalized for COVID-19 and treated with remdesivir or placebo for 5 days. Out of 243 participants enrolled and treated in the study, 42 participants had solid organ transplantation (SOT) and received immunosuppressive medication prior to and during the study. Nasopharyngeal swabs were collected at baseline and through Day 29. Virology assessments were performed using SARS-CoV-2 RT-qPCR, next-generation sequencing, and site-directed mutagenesis in a SARS-CoV-2 replicon system. Remdesivir treatment demonstrated significantly greater reductions from baseline in viral load on Day 5 and Day 7 compared with placebo (*P* ≤ 0.034). In the remdesivir treatment group, participants with SOT showed significantly smaller viral load reductions from baseline on Days 5, 7, and 14 compared with participants without SOT (all *P* < 0.05). Mathematical modeling of viral load kinetics in participants with kidney transplants predicted a >13-day faster time to viral load decline by extending the remdesivir treatment duration from 5 days to 10 days. In four participants treated with remdesivir, emergent substitutions in Nsp12 (E136V, M794I, and C799F) with low-level reduced *in vitro* susceptibility to remdesivir (≤3.4 fold change in half-maximal effective concentration) were observed 9 days after cessation of remdesivir treatment. Three of the four participants had undergone SOT. Overall, remdesivir demonstrated antiviral activity in participants with impaired kidney function, with delayed viral clearance in SOT recipients and rare posttreatment emergence of Nsp12 substitutions that confer low-level reduced susceptibility to remdesivir.

**CLINICAL TRIALS:**

This study is registered with ClinicalTrials.gov as NCT04745351; EudraCT registration no. 2020-005416-22.

## INTRODUCTION

Remdesivir is a nucleotide analog prodrug whose active metabolite inhibits the RNA-dependent RNA polymerase (RdRp) complex of multiple viruses, including SARS-CoV-2 (1–5). The target of remdesivir within coronaviruses is the nonstructural protein Nsp12, which contains the RdRp active site (5–8). Studies have shown that remdesivir retains potent antiviral activity against all major SARS-CoV-2 variants of concern, including Delta and Omicron (9–11).

Remdesivir is approved for the treatment of COVID-19 in hospitalized and nonhospitalized adults and pediatric patients (12). Randomized controlled clinical studies have shown that treatment with remdesivir is associated with a shorter time to recovery and improved clinical status compared with standard of care alone in hospitalized patients with COVID-19 (13, 14) and prevention of progression from mild to severe COVID-19 in nonhospitalized high-risk patients (15).

The Phase 3 REDPINE study evaluated the efficacy and safety of remdesivir in individuals with impaired kidney function who were hospitalized for COVID-19. Although underpowered for the assessment of efficacy, REDPINE demonstrated the safety of remdesivir for the treatment of COVID-19, including for those on dialysis, without the requirement for dose adjustment (16). This study supported the decision of the US Food and Drug Administration to expand the label for this population (17). REDPINE enrolled participants with and without solid organ transplantation (SOT), and several participants received immunosuppressive drugs prior to and during the study.

Prolonged shedding of SARS-CoV-2 has been documented in viral dynamics studies in people with immunocompromising conditions, including SOT recipients (18–20). Studies suggest that viral shedding may persist for more than 1 month (20), which may necessitate alternative vaccination and treatment strategies to decrease the duration of shedding. The EPIC-IC trial, which evaluated an extended nirmatrelvir-ritonavir treatment duration for nonhospitalized immunocompromised patients, found that the median time to reach a sustained viral load below the lower limit of quantification (LLOQ) was shorter after 10 days of treatment (13 days to reach LLOQ) compared with 5 days of treatment (28 days to reach LLOQ) in severely immunocompromised participants (21). Severely immunocompromised participants who received 5 days of nirmatrelvir-ritonavir treatment were less likely to remain symptom-free and SARS-CoV-2 negative compared with those who received 10 or 15 days of treatment.

In virologic resistance analyses from the ACTT-1, SIMPLE, and PINETREE phase 3 clinical trials, emergent substitutions in Nsp12 occurred in a small percentage of participants receiving remdesivir or placebo (22–24). Substitutions associated with reduced susceptibility to remdesivir were identified in 2 of 31 participants in the remdesivir arm of ACTT-1 and in 1 of 115 participants in PINETREE; none were identified in the SIMPLE trial.

Here, we present virology analyses from the REDPINE study, including viral load dynamics in participants with and without SOT, and resistance analyses. Mathematical modeling was used to estimate the effect of extending remdesivir treatment beyond 5 days on the time to viral clearance in kidney transplant recipients.

## RESULTS

### Participants

**Fig 1:**
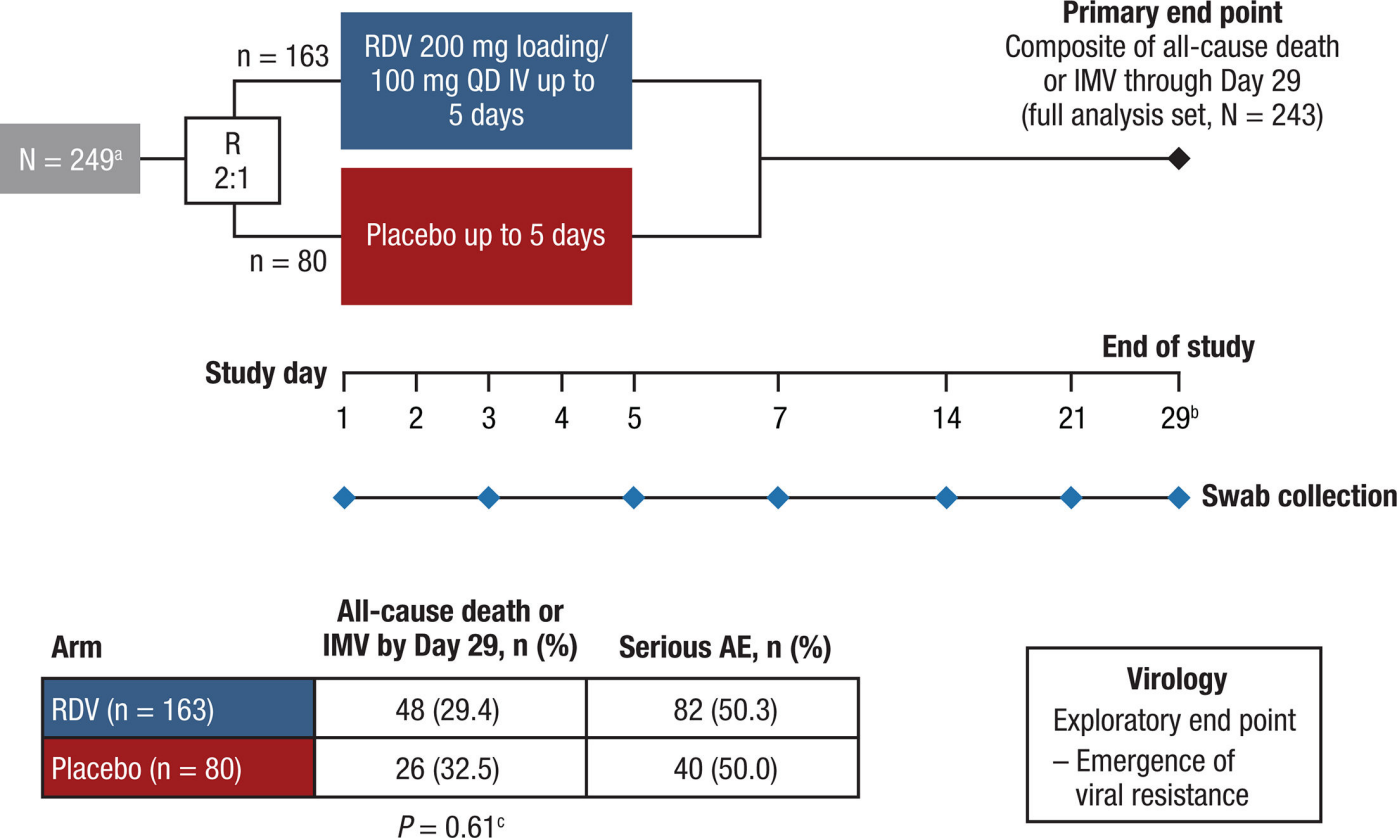
REDPINE study design. ^a^249 participants were randomized, but six were not treated. ^b^If a participant was discharged prior to Day 29, a phone follow-up was completed on Days 29 and 60. ^c^The study was underpowered for efficacy due to insufficient enrollment. AE, adverse event; IMV, invasive mechanical ventilation; IV, intravenous; QD, once daily; R, randomization; RDV, remdesivir.

The REDPINE study enrolled and treated 243 participants (remdesivir, *n* = 163; placebo, *n* = 80; Fig. 1). Of the 243 participants, 42 (remdesivir, 35 [21%]); placebo, 7 [9%]) had a history of SOT and received immunosuppressive drugs prior to and during the study. Of these, 34 (remdesivir, 28 [80%]; placebo, 6 [86%]) participants had a history of kidney transplantation. The baseline characteristics and clinical outcomes of this study have been published previously (16). The mean (SD) age was 68 (14) years among participants receiving remdesivir and 71 (13) years among participants receiving placebo. The mean (SD) body mass index was 29.5 (7.1) kg/m^2^ among participants receiving remdesivir and 28.9 (6.0) kg/m^2^ among participants receiving placebo.

### Viral load analyses

**Fig 2:**
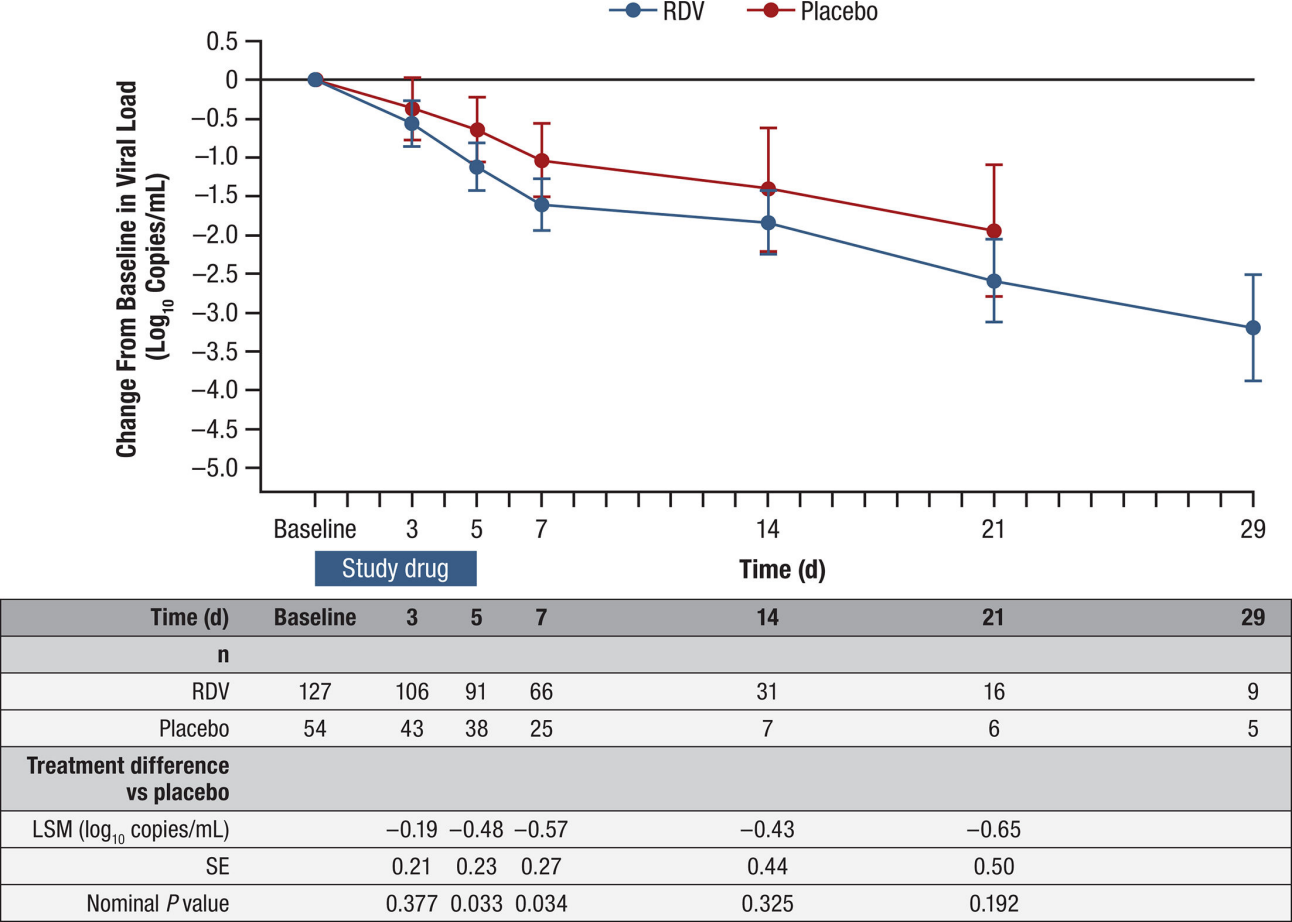
LSM (95% CI) change from baseline in the SARS-CoV-2 viral load by the study drug (Virology Analysis Set). LSM change from baseline in the viral load and *P* values were determined using MMRM. Virology Analysis Set included all participants who were randomized into the study, received ≥1 dose of the study drug, and had positive SARS-CoV-2 at baseline. CI, confidence interval; LSM, least-squares mean; MMRM, mixed model for repeated measures; RDV, remdesivir; SARS-CoV-2, severe acute respiratory syndrome coronavirus 2; SE, standard error.

The observed mean SARS-CoV-2 viral load at baseline was similar between the remdesivir and placebo groups (Table 1). Viral load analyses demonstrated that remdesivir treatment led to significantly greater reductions from baseline in viral load on Day 5 and Day 7 compared with placebo. At Day 5, the least-squares mean (SE) change from baseline in viral load was –1.12 (0.16) log_10_ copies/mL for the remdesivir group and –0.64 (0.21) log_10_ copies/mL for the placebo group, with a least-squares mean difference of –0.48 log_10_ copies/mL (95% CI, –0.93 to –0.04; *P* = 0.033; Fig. 2). At Day 7, the least-squares mean change from baseline in viral load was –1.61 (0.17) log_10_ copies/mL for the remdesivir group and –1.04 (0.24) log_10_ copies/mL for the placebo group, with a least-squares mean difference of −0.57 log_10_ copies/mL (95% CI, –1.09 to –0.04; *P* = 0.034; Fig. 2).

**TABLE 1:** SARS-CoV-2 viral load (log_10_ copies/mL) at baseline (Virology Analysis Set^*a*^)

	Mean (SD)	Median (Q1, Q3)
Remdesivir (*n* = 127)	5.9 (1.5)	6.2 (4.9, 7.0)
SOT recipients (*n* = 29)	6.5 (1.2)	6.4 (6.3, 7.1)
Kidney transplant recipients (*n* = 23)	6.4 (1.3)	6.4 (6.0, 7.1)
Non–SOT recipients (*n* = 98)	5.8 (1.5)	5.8 (4.5, 6.9)
Placebo (*n* = 54)	6.2 (1.5)	6.1 (5.0, 7.5)

^*a*^ Virology analysis set included all participants who were randomized into the study, received ≥1 dose of the study drug, and had positive SARS-CoV-2 RT-qPCR at baseline. Q1, first quartile; Q3, third quartile; RT-qPCR, quantitative reverse transcriptase–polymerase chain reaction; SD, standard deviation; SOT, solid organ transplantation.

### Viral load analyses in participants with SOT

**Fig 3:**
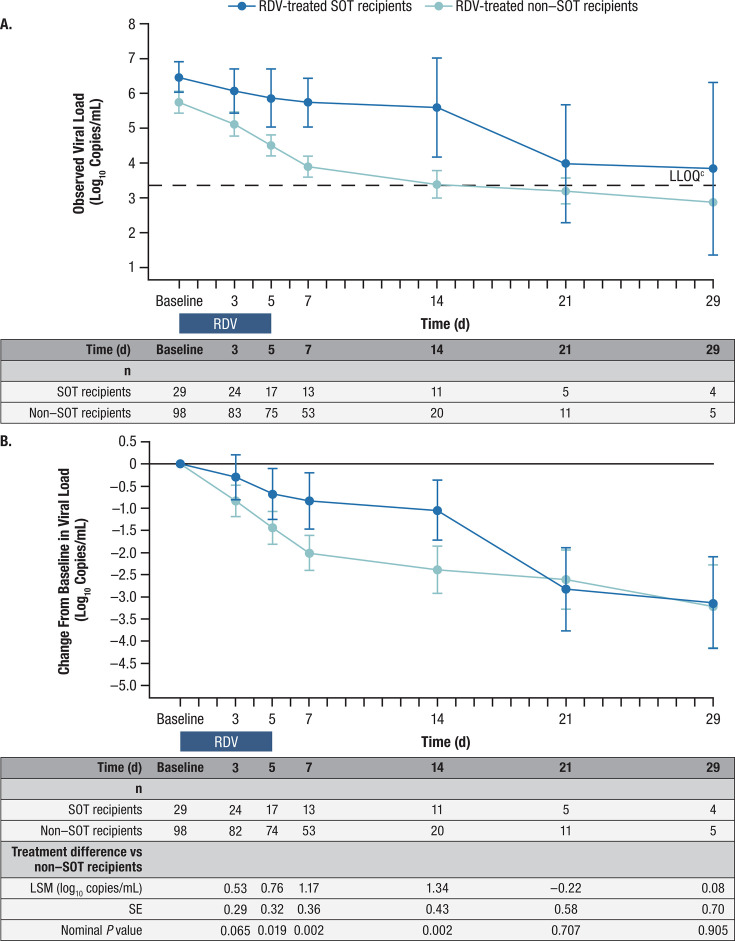
Observed mean (SD) (**A**) and LSM (95% CI) change from baseline (**B**) in SARS-CoV-2 viral load among remdesivir-treated participants by SOT status (Virology Analysis Set). LSM change from baseline in viral load and *P* values were determined using MMRM. Virology Analysis Set included all participants who were randomized into the study, received ≥1 dose of the study drug, and had positive SARS-CoV-2 at baseline. The LLOQ of the RT-qPCR viral load assay was 3.35 log_10_ copies/mL. CI, confidence interval; LLOQ, lower limit of quantification; LSM, least-squares mean; MMRM, mixed model for repeated measures; RT-qPCR, quantitative reverse transcriptase–polymerase chain reaction; SD, standard deviation; SE, standard error; SOT, solid organ transplantation; RDV, remdesivir.

Among remdesivir-treated participants, the observed mean SARS-CoV-2 viral load was higher in SOT recipients compared with non–SOT recipients from baseline through Day 29 (Fig. 3A). When comparing SOT recipients and non–SOT recipients, 3/11 (27%) versus 14/20 (70%), respectively, reached the LLOQ for the quantitative reverse transcriptase–polymerase chain reaction (RT-qPCR) viral load assay on Day 14. Remdesivir-treated SOT recipients had significantly smaller viral load reductions from baseline on Days 5, 7, and 14 compared with remdesivir-treated non–SOT recipients (all *P* < 0.05; Fig. 3B). In participants with SOT, remdesivir treatment led to greater reductions from baseline in viral load on Days 3, 5, and 7 compared with placebo; however, these differences were not statistically significant (Table S1).

### Viral load analysis in participants with a history of kidney transplantation

**Fig 4:**
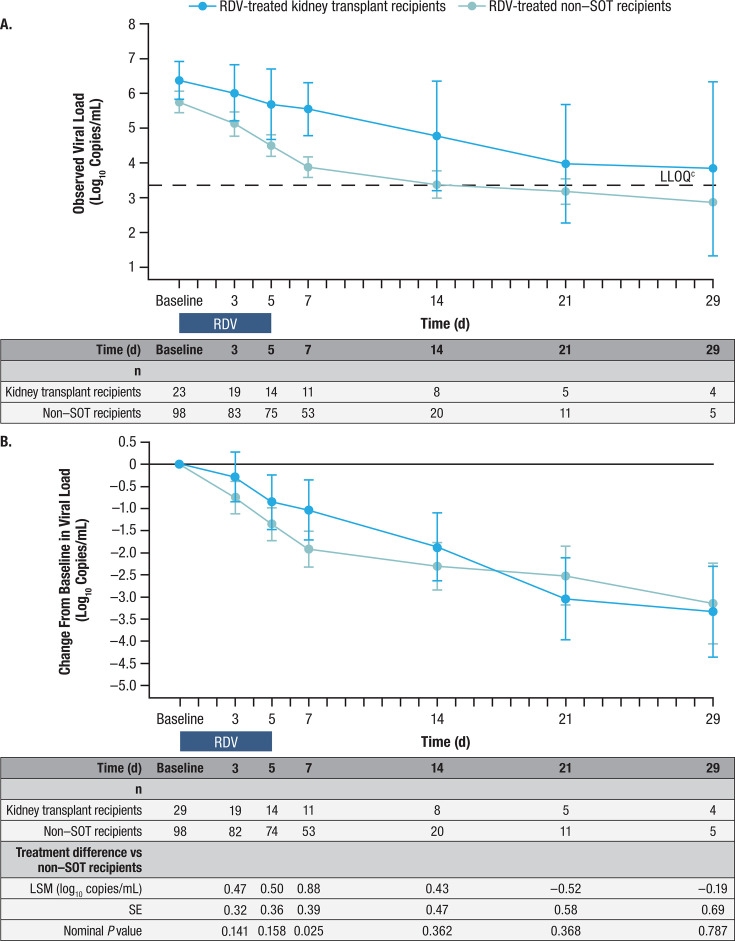
Observed mean (SD) (**A**) and LSM (95% CI) change from baseline (**B**) in SARS-CoV-2 viral load among remdesivir-treated participants by the kidney transplant status (Virology Analysis Set). LSM change from baseline in viral load and *P* values were determined using MMRM. Virology Analysis Set included all participants who were randomized into the study, received ≥1 dose of the study drug, and had positive SARS-CoV-2 at baseline. The LLOQ of the RT-qPCR viral load assay was 3.35 log_10_ copies/mL. CI, confidence interval; LLOQ, lower limit of quantification; LSM, least-squares mean; MMRM, mixed model for repeated measures; RDV, remdesivir; RT-qPCR, quantitative reverse transcriptase–polymerase chain reaction; SD, standard deviation; SE, standard error; SOT, solid organ transplantation.

The observed mean SARS-CoV-2 viral load among remdesivir-treated participants was higher in kidney transplant recipients compared with non–SOT recipients from baseline through Day 29 (Fig. 4A). When comparing kidney transplant recipients and non–SOT recipients, 3/8 (38%) versus 14/20 (70%), respectively, reached the LLOQ for the RT-qPCR viral load assay on Day 14. Kidney transplant recipients had smaller reductions from baseline in viral load compared with non–SOT recipients, with a statistically significant difference on Day 7 (least-squares mean [LSM] difference, 0.88 log_10_ copies/mL; *P* = 0.025; Fig. 4B).

### Impact of treatment duration on SARS-CoV-2 viral load decline

**Fig 5:**
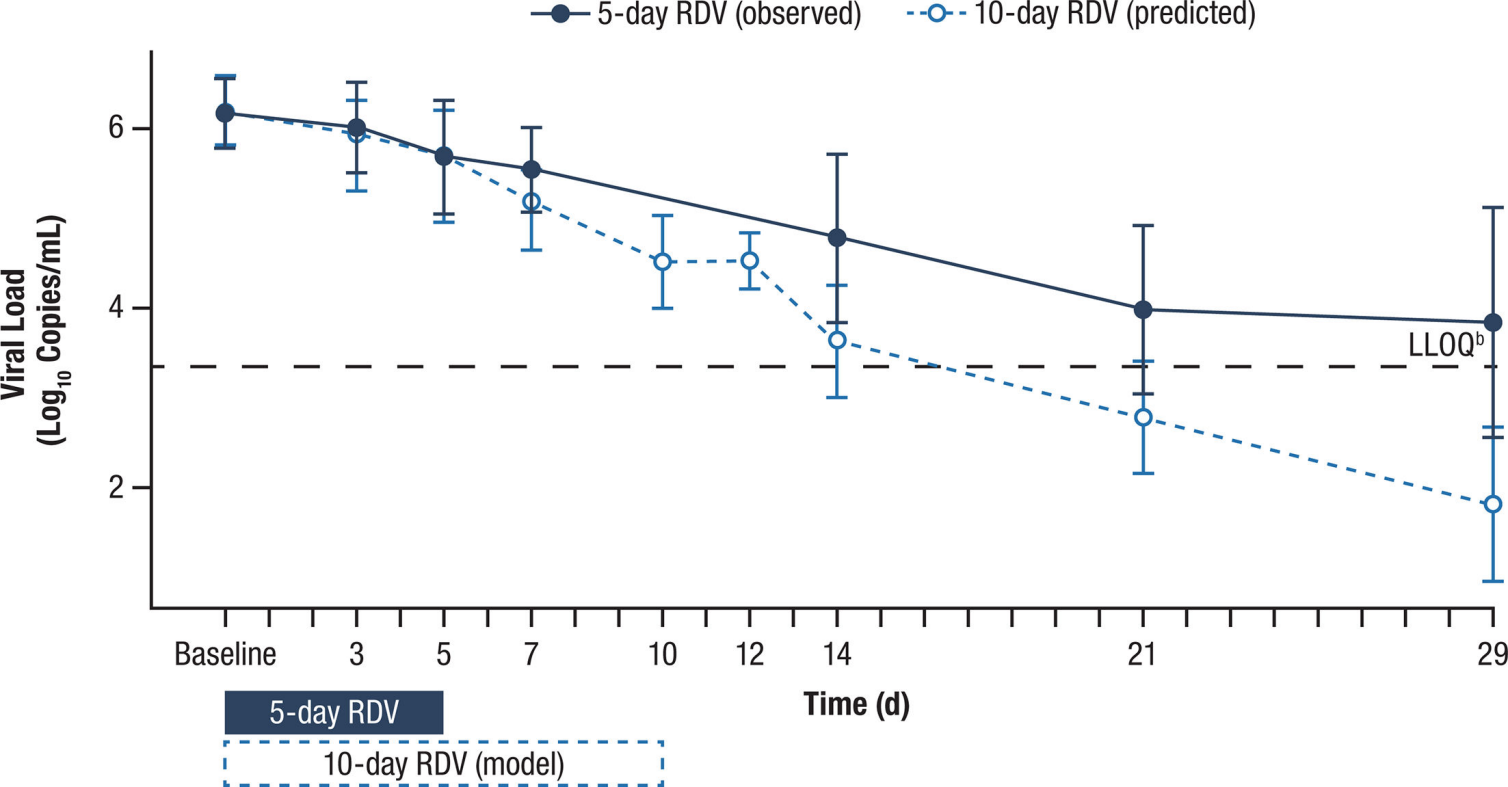
Predicted mean (95% CI) SARS-CoV-2 viral load over time with a 5-day versus 10-day remdesivir treatment duration^a^ in kidney transplant recipients. ^a^Predicted mean (95% CI) viral load with a 10-day RDV treatment duration was predicted using a linear mixed-effects model. ^b^The LLOQ of the RT-qPCR viral load assay was 3.35 log_10_ copies/mL. CI, confidence interval; LLOQ, lower limit of quantification; RDV, remdesivir; RT-qPCR, quantitative reverse transcriptase–polymerase chain reaction.

In the REDPINE study, the remdesivir treatment duration was 5 days. A linear mixed-effects model was used to predict the effect of extending the remdesivir treatment duration on viral load dynamics. Only those participants with a history of kidney transplantation (23/29 remdesivir-treated SOT recipients) were included in the model, as participants with other transplants did not have data after Day 14. The mean viral load in kidney transplant recipients receiving a 10-day remdesivir treatment duration was predicted by fitting baseline to Day 5 viral load data to an on-treatment model and post–Day 5 viral load change data to a posttreatment model (Fig. 5). The model predicted that the mean viral load would reach the LLOQ >13 days faster with a 10-day remdesivir treatment duration (mean viral load <LLOQ at Day 16) versus a 5-day remdesivir treatment duration (mean viral load >LLOQ at Day 29).

### Resistance analyses

Nasopharyngeal samples from participants were eligible for virologic resistance analyses if there was all-cause death or invasive mechanical ventilation (IMV), or if SARS-CoV-2 viral load at Day 14 was higher than the LLOQ. Of the 243 participants who were treated in the REDPINE study, 82 met these criteria (55 in the remdesivir group and 27 in the placebo group; Table 2). Of these, 15 were excluded from the resistance analyses due to having no viral load data at baseline and/or all post-baseline time points or because the viral load was <LLOQ at baseline. Sequencing was attempted on all baseline and post-baseline samples from the remaining 67 participants.

**TABLE 2:** Participants included in the virologic resistance analyses

Treated, *n* (%)	Remdesivir (*n* = 163)	Placebo (n = 80)	Total (*n* = 243)
Participants who qualified for resistance analyses	55 (33.7)	27 (33.8)	82 (31.2)
Participants with all-cause death or IMV	48 (29.4)	26 (32.5)	74 (28.1)
Participants with SARS-CoV-2 viral load >LLOQ at baseline and ≥1 post-baseline time point	39 (23.9)	20 (25.0)	59 (22.4)
Participants with SARS-CoV-2 viral load >LLOQ at Day 14	18 (11.0)	5 (6.3)	23 (8.7)
Participants with SARS-CoV-2 viral load >LLOQ at Day 14 without death or IMV and baseline viral load >LLOQ	7 (4.3)	1 (1.3)	8 (3.0)
Participants with sequencing attempted^a^	46 (28.2)	21 (26.3)	67 (25.5)
Participants with baseline data available^b^	45 (27.6)	20 (25.0)	65 (24.7)
Participants with post-baseline data available^c^	42 (25.8)	20 (25.0)	62 (23.6)
Participants with both baseline and post-baseline data available	41 (25.2)	19 (23.8)	60 (22.8)

^a^ Sequencing was not attempted for the participants who qualified for resistance analyses but had no baseline and/or post-baseline viral load data (nine participants), insufficient baseline viral load (five participants) or unavailable viral load data (one participant).

^b^ Samples from two participants (one in each of the remdesivir and placebo groups) were erroneously labeled Day 1 and tested but did not correspond to the first day of treatment and were excluded from baseline resistance analysis.

^c^ Post-baseline sequence data were not available for four participants in the remdesivir group and one participant in the placebo group due to assay failure. IMV, invasive mechanical ventilation; LLOQ, lower limit of quantification.

The most prevalent SARS-CoV-2 lineages at baseline were BA.1.1 (Omicron; 19% of participants), AY.43 (Delta; 11%), and AY.103 (Delta; 9%). All participants with sequencing data had the Nsp12 P323L substitution present at baseline. In 38 (58%) participants, the Nsp12 G671S substitution was also observed.

### Emergent amino acid substitutions

Among the 67 participants for whom sequencing was attempted, baseline and post-baseline sequencing data were available for a total of 60 (90%) participants (41 in the remdesivir group and 19 in the placebo group; Table 2). Emergent substitutions in Nsp12 were detected in nine participants (eight in the remdesivir group and one in the placebo group; Table 3). Eight different substitutions were detected in the participants from the remdesivir group: F694Y in three participants, M794I in two participants, and G44V and C799F in one participant each; one participant had both E136K and T801I substitutions present, and another participant had both E136V and I223M substitutions present. All substitutions emerged 9 days after cessation of remdesivir treatment (Day 14), except the F694Y substitution, which was detected in two participants on Day 5 (i.e., the last day of remdesivir treatment).

**TABLE 3:** Emergent amino acid substitutions detected in Nsp12 at post-baseline

	Participants with both baseline and post-baseline sequencing data available, *n* (%)		
Emergent Nsp12 substitutions	Remdesivir (*n* = 41)	Placebo (*n* = 19)	Total (*n* = 60)
Any emergent substitutions	8 (19.5)	1 (5.3)	9 (15.0)
G44V	1 (2.4)	0	1 (1.7)
E136K + Y253H^a^ + T801I	1 (2.4)	0	1 (1.7)
E136V + I223M	1 (2.4)	0	1 (1.7)
P384P	0	1 (5.3)	1 (1.7)
F694Y	3 (7.3)	0	3 (5.0)
M794I	2 (4.9)	0	2 (3.3)
C799F	1 (2.4)	0	1 (1.7)

^a^ Y253H was an emergent substitution in Nsp13.

**Fig 6:**
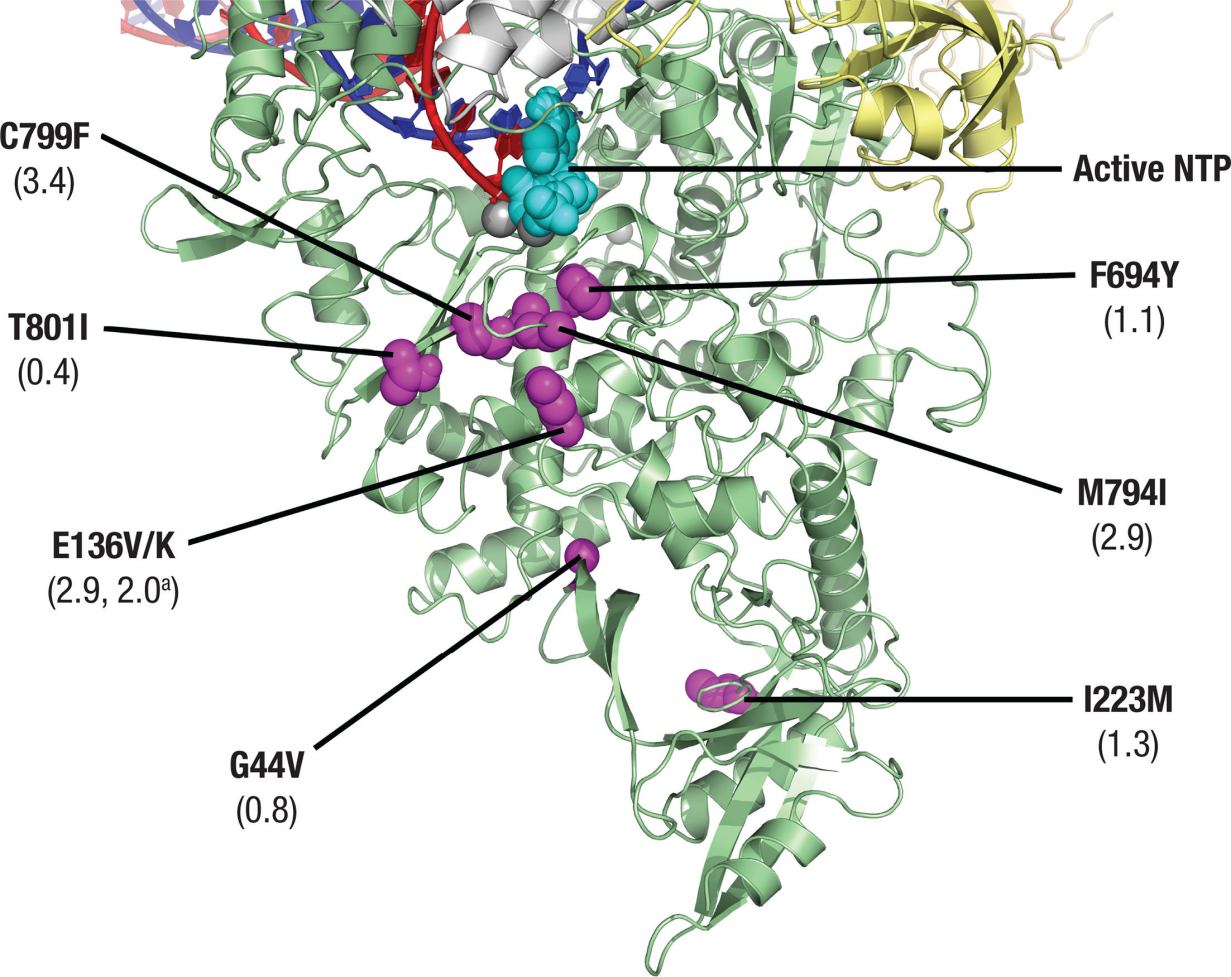
Map of remdesivir-emergent Nsp12 substitutions on a model of the SARS-CoV-2 polymerase complex derived from cryo-electron microscopy structures. EC_50_ fold change values versus wild-type are listed in parentheses for each substitution. ^a^EC_50_ fold change values versus wild-type for E136V and E136K are 2.9 and 2.0, respectively. EC_50_, half-maximal effective concentration; NTP, nucleoside triphosphate.

The structural impact of the six Nsp12 substitutions that emerged in the remdesivir group was analyzed based on a model derived from two cryo-electron microscopy structures of the SARS-CoV-2 polymerase complex (Fig. 6) (8, 25). In this model, none of the substitutions were in direct contact with the incoming nucleoside triphosphate (NTP) substrate or the RNA. However, F694Y was close to the NTP substrate, and M794I, C799F, and T801I were part of Motif D of Nsp12, which governs the closing of the active site. Nsp12 E136K and E136V were in direct contact with this motif.

Six emergent substitutions in Nsp13 were detected in participants treated with remdesivir: V6F, T127I, E136D, Q194*, Y253H, and Y541C, where “*” indicates that the substitution resulted in a stop codon. No emergent substitutions in Nsp8, Nsp10, or Nsp14 were detected.

### Phenotypic analysis of emergent amino acid substitutions in the remdesivir group

Replicons containing the emergent Nsp12 substitutions in the remdesivir group were compared with replicons containing the wild-type Nsp12 (SH01). The replicons containing the G44V and I223M substitutions had 0.8- and 1.3-fold changes in effective concentration of remdesivir to reach 50% inhibition of virus replication (EC_50_), respectively, indicating full susceptibility to remdesivir (Table 4). The replicon containing the E136K + Nsp13 Y253H + T801I substitutions also demonstrated full susceptibility to remdesivir, with a 1.9-fold change in EC_50_. The remainder of the replicons, containing the E136V, M794I, and C799F amino acid substitutions, had fold changes of 2.9 to 3.4 in EC_50_, indicating low-level reduced susceptibility to remdesivir. The F694Y substitution was tested in the replicon assay but did not replicate and was therefore tested using a recombinant SARS-CoV-2 (Wuhan-Hu-1, NC_045512.2 [WA1]) virus containing a nanoluciferase transgene, as in a previous study (26). This showed a 1.1-fold change in EC_50_ versus wild-type virus, indicating no impact on remdesivir susceptibility. Full EC_50_ curves for emergent Nsp12 substitutions are presented in Fig. S1.

**TABLE 4:** Remdesivir EC_50_ against SARS-CoV-2 reference and Nsp12 mutants

Nsp12 substitution (*N*^*a*^)	Mean (SD) remdesivir EC_50_, nM	EC_50_ fold change from wild-type^*b*^	Replication capacity versus wild-type,^*c*^ %	Testing system	Frequency,^*d*^ % (*n*)
SH01 wild-type (8)	9.5 (0.5)	1.0	100	Replicon	
V792I (positive control) (6)	28.2 (2.0)	3.0	9-305^*e*^	Replicon	0.00461 (795)
G44V (2)	8.0 (0.9)	0.8	32	Replicon	0.01000 (1706)
E136K (4)	21.0 (4.4)	2.0	6	Replicon^*f*^	0.00010 (20)
E136K + Y253H^*g*^ + T801I (2)	20.1 (2.2)	1.9	88	Replicon^*f*^	–^*j*^
T801I (2)	3.7 (0.3)	0.4	36	Replicon^*f*^	0.01000 (2400)
E136V (2)	27.2 (1.2)	2.9	219	Replicon	0.00015 (25)
E136V + I223M (2)	27.2 (1.8)	2.9	254	Replicon	–^*j*^
I223M (2)	12.1 (0.3)	1.3	124	Replicon	0.01000 (2530)
F694Y (2)	112.3 (1.6)	1.1	NA	Recombinant^*h*^	1.90000 (324,580)
M794I (2)	27.6 (1.3)	2.9	388	Replicon	0.00362 (624)
C799F (2)	49.9 (2.5)	3.4	35	Replicon^*i*^	0.00134 (231)

^*a*^ Number of times the experiment was performed for each substitution.

^*b*^ EC_50_ fold change for each mutant was calculated relative to the SH01 wild-type reference strain in the presence of remdesivir. The cutoff for *in vitro* susceptibility, which was based on the assay variability, was 2.5-fold change.

^*c*^ Replication capacity of the mutant replicon was calculated by dividing the maximum luciferase signal of the mutant replicon by that of the wild-type replicon at 48 h post-infection.

^*d*^ Frequency was calculated based on >17 million SARS-CoV-2 sequences in the GISAID database as of 2 February 2026.

^*e*^ Range of reported replication capacity values for V792I (22, 27 ).

^*f*^ E136K and T801I single substitutions and the E136K + Y253H + T801I triple substitution were tested separately in the replicon assay, and the EC_50_ fold changes were calculated using the wild-type replicon from that experiment, with a mean ± SD EC_50_ value of 10.6 ± 4.0 nM.

^*g*^ Y253H was an emergent substitution in Nsp13. Mean (SD) remdesivir EC_50_ and EC_50_ fold change from wild-type for Y253H as a single substitution can be found in Table S2. The frequency (*n*) of the Y253H substitution in the GISAID database was 0.04000% (6,839).

^*h*^ F694Y was tested using a recombinant SARS-CoV-2 WA1 virus containing a nanoluciferase transgene. EC_50_ fold change was calculated from the WA1 wild-type strain, with a mean ± SD EC_50_ value of 99 ± 2 nM. F694Y was tested in the replicon assay and did not replicate (26).

^*i*^ C799F was tested separately in the replicon assay, and the EC_50_ fold-change value was calculated using the wild-type replicon from that experiment, with a mean ± SD EC_50_ value of 14.7 ± 3.0 nM. EC_50_, half-maximal effective concentration; GISAID, Global Initiative on Sharing All Influenza Data; NA, not applicable; SD, standard deviation.

^*j*^ –, combination mutant frequencies were not obtained from the GISAID database.

SARS-CoV-2 replicons containing Nsp12 G44V and Nsp12 C799F substitutions demonstrated replication capacities of 32% and 35% relative to wild-type, respectively (Table 4). A replicon containing Nsp12 E135K + Nsp13 Y253H + Nsp12 T801I demonstrated a replication capacity of 88% relative to wild-type. All other mutant replicons demonstrated replication capacities between 6% and 388% of wild-type.

With respect to the emergent Nsp13 substitutions, all demonstrated a ≤2.3-fold change in EC_50_, indicating no reduction in susceptibility to remdesivir (Table S2). The Nsp13 Q194* substitution was not phenotypically analyzed, as this mutation would produce a stop codon and likely result in a dead virus. Two additional Nsp13 substitutions, S485L, which was detected below the 15% cutoff for variant reporting, and Y253H, which was detected in combination with Nsp12 E136K + T801I, were included in the phenotypic analysis and showed similar susceptibility to remdesivir compared with the wild-type replicon.

An analysis of publicly available SARS-CoV-2 sequences in the Global Initiative on Sharing All Influenza Data (GISAID) database demonstrated that F694Y was the most common emergent Nsp12 substitution, occurring in 1.9% of >17 million sequences available as of 2 February 2026. All other substitutions occurred at a frequency <0.05% (Table 4).

### Characteristics of participants with emergent Nsp12 substitutions conferring reduced susceptibility to remdesivir

**Fig 7:**
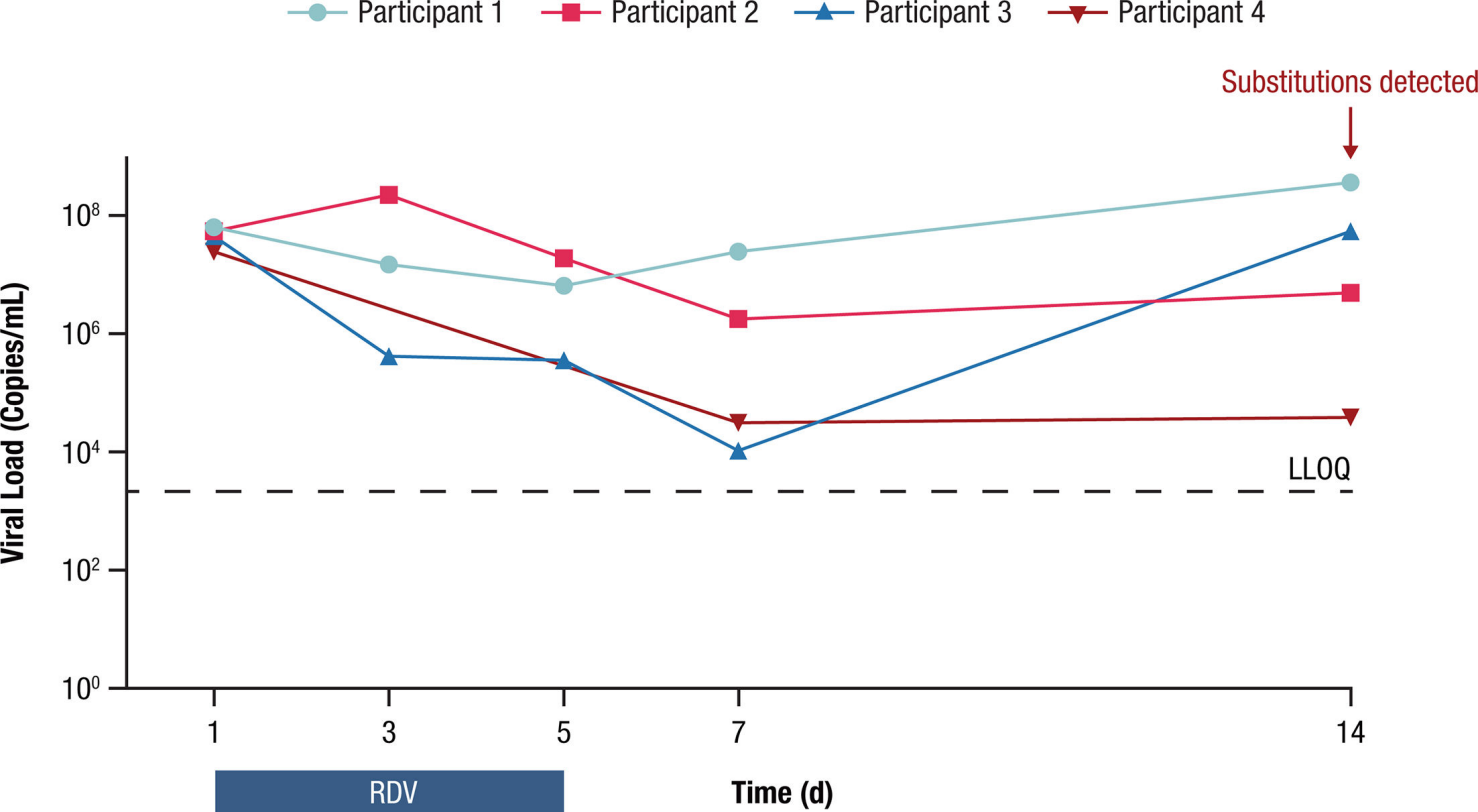
Viral load dynamics in participants with Nsp12 substitutions associated with reduced susceptibility to remdesivir. LLOQ, lower limit of quantitation; RDV, remdesivir.

Four participants had emergent substitutions with low-level reduced susceptibility to remdesivir; viral load dynamics for these participants are presented in Fig. 7. All experienced a viral load decline while receiving remdesivir, and no substitutions were detected at Day 5 (the last day of remdesivir treatment).

These participants had varying levels of kidney disease and other risk factors for progression to severe COVID-19. Three of these four (75.0%) participants had undergone SOT and were on concomitant immunosuppressive therapies compared with 35 of 163 (21.5%) remdesivir-treated participants in the overall study population. The emergent substitutions were present as mixtures with the wild-type amino acid and were detected in the samples collected 9 to 10 days after the cessation of remdesivir treatment (Fig. 7).

In participant 1, who had chronic kidney disease, hypertension, cardiovascular/cerebrovascular disease, and diabetes and had received a lung transplant (virologic resistance analysis criteria: all-cause death or IMV by Day 29), viral load decreased from 63,318,663 copies/mL at baseline to 6,473,055 copies/mL at Day 5 of remdesivir treatment but increased to 365,366,125 copies/mL at Day 14. The M794I substitution was first detected at Day 14 (9 days after remdesivir cessation) with a substitution frequency of 64.6%. The participant experienced no improvement from baseline in clinical status (hospitalized and requiring supplemental oxygen) and died at Day 18.

Participant 2 had kidney failure, chronic lung disease, hypertension, and had received a kidney transplant (virologic resistance analysis criteria: viral load >LLOQ on Day 14). Their viral load decreased from 54,577,850 copies/mL at baseline to 18,985,892 copies/mL at Day 5 and further to 4,898,722 copies/mL at Day 14 (9 days after remdesivir cessation), when the M794I substitution was detected (15.6% substitution frequency). The participant’s clinical status had improved by Day 65 (no longer required hospitalization).

Participant 3 had kidney failure, hypertension, cardiovascular/cerebrovascular disease, diabetes, body mass index [BMI] ≥ 30 kg/m^2^, and current cancer and had received a kidney transplant (virologic resistance analysis criteria: viral load >LLOQ on Day 14); the kidney transplant was a failed transplant, with the participant returning to dialysis. Their viral load decreased from 45,209,641 copies/mL at baseline to 360,797 copies/mL at Day 5 of remdesivir treatment but increased to 54,455,650 copies/mL at Day 14 (9 days after remdesivir cessation), when the C799F substitution was detected (15.7% substitution frequency), with worsening clinical status. Their clinical status had improved by Day 64 (no longer required hospitalization).

Participant 4 had acute kidney injury (AKI), BMI ≥ 30 kg/m^2^, and chronic liver disease (virologic resistance analysis criteria: all-cause death or IMV by Day 29). Their viral load decreased from 24,452,105 copies/mL at baseline to 31,246 copies/mL at Day 7 (data were not available at Day 5) but increased to 38,658 copies/mL at Day 14 (10 days after remdesivir cessation), when the I223M and E136V substitutions were first detected, with 56.2% and 22.1% substitution frequencies, respectively. The participant’s clinical status initially improved but subsequently deteriorated, and the participant died on Day 28.

## DISCUSSION

In remdesivir-treated participants with SOT in this study, viral load reductions were slower compared with participants without SOT, potentially due to diminished immune system support to clear the virus. Our modeling of the viral load data from participants with kidney transplants predicted that a 10-day remdesivir treatment would result in a faster reduction of viral load, with the mean viral load reaching the LLOQ by Day 16, while the mean viral load for participants who received a 5-day treatment was still above the LLOQ at Day 29. Due to known prolonged viral shedding in immunocompromised patients (18, 19), such individuals might benefit from an antiviral treatment duration longer than 5 days, as supported by the findings of the current study.

Among hospitalized participants who received 5 days of remdesivir treatment, the mean viral load reached the LLOQ at Day 14 in non–SOT recipients, while the mean viral load in SOT recipients, including kidney transplant recipients, remained above the LLOQ at Day 29. This is similar to the viral load results of the nonhospitalized, severely immunocompromised participants, including individuals with SOT, who received 5 days of nirmatrelvir-ritonavir treatment from the EPIC-IC study (21). The EPIC-IC study showed that the viral load remained above the LLOQ at Day 28 and beyond for several severely immunocompromised participants following 5 days of nirmatrelvir-ritonavir treatment, similar to the results observed in the SOT recipients who received a 5-day remdesivir treatment course in the current study.

In this study, eight unique emergent Nsp12 substitutions were observed in participants in the remdesivir group. Of these, three substitutions (E136V, M794I, and C799F) were associated with low-level *in vitro* reduced susceptibility to remdesivir (EC_50_ fold changes of 2.9-, 2.9-, and 3.4-fold, respectively); the remaining substitutions (G44V, E136K + T801I, I223M, and F694Y) were not associated with reduced susceptibility to remdesivir. Two substitutions that emerged, F694Y and C799F, have previously been detected in Phase 3 clinical studies or resistance selection experiments. Similarly to previous cases, the emergent F694Y substitution has previously been detected in three participants (one treated with remdesivir and two treated with placebo) in a Phase 3 clinical trial (24) and, during *in vitro* resistance selection experiments, was not associated with reduced susceptibility to remdesivir (26), which is consistent with this report. The emergent C799F substitution, which was associated with low-level reduced susceptibility (3.4-fold change in EC_50_), was previously observed in the virologic resistance analyses of a Phase 3 clinical trial in one participant whose clinical status gradually improved over time (22). Of the other emergent Nsp12 substitutions detected during the current study, most have not been detected in other virologic resistance analyses of Phase 3 clinical trial data of remdesivir (22–24) or in resistance selection experiments (28–30); G44V and M794I have each been reported separately in one patient following treatment with remdesivir (22, 31). Of note, two emergent Nsp12 substitutions (E136V and M794I) demonstrated increased replication capacity compared with wild-type, raising the possibility of enhanced viral fitness in these variants; however, these substitutions have only been observed at low frequencies in the GISAID database (≤0.00362%). Importantly, the substitutions with reduced susceptibility emerged after cessation of remdesivir treatment and were not detected during active therapy. The clinical impact of the emergence of these substitutions is unclear.

Three out of four participants with emergent substitutions associated with low-level reduced susceptibility to remdesivir had previously undergone SOT and were receiving concomitant immunosuppressive therapies. A small number of case studies in immunosuppressed patients with prolonged SARS-CoV-2 shedding have identified the emergence of Nsp12 substitutions during treatment with remdesivir (31–34). Some of these substitutions, including V792I and E802D, have been associated with low-level reduced susceptibility to remdesivir *in vitro* (31, 32).

There are key limitations to this analysis, particularly the low percentage of participants who had both baseline and post-baseline samples available (60 of 82 participants who qualified for resistance analyses) and the limited number of participants with SOT overall and in the resistance analysis subcohort. Furthermore, the number of participants with SOT in the placebo group was relatively low, which added uncertainty to the mixed model repeated measures (MMRM) viral load analysis comparing remdesivir with placebo within the SOT population. In addition, the predictive modeling was based on viral load data from a 5-day treatment period and did not include 10-day treatment data. Only kidney transplant recipients were included in the mathematical model of extended remdesivir treatment due to limited data for participants with other types of SOT. Because the current analysis was limited to individuals with renal impairment and immunosuppression associated with SOT, the findings of this study may not be generalizable to individuals with other types of immunocompromising conditions, such as those with hematologic-oncologic diseases and primary immunodeficiencies. However, the findings from this study provide a robust analysis of viral kinetics in a relatively homogenous population of SOT recipients with renal impairment, a group for which data are limited. Moreover, the results of this study support those of the previous analyses from other Phase 3 clinical trials of remdesivir in patients with COVID-19, which demonstrated a high barrier to the development of resistance to remdesivir (22–24).

In conclusion, the viral load analyses from this Phase 3 clinical trial show that, while remdesivir treatment reduced SARS-CoV-2 viral load compared with placebo in participants overall, testing an extended duration of remdesivir treatment may be beneficial in immunocompromised populations, such as SOT recipients, to mitigate prolonged viral shedding. However, additional studies in individuals with other types of immunocompromising conditions are needed to confirm the potential benefit of prolonged remdesivir treatment. The resistance analyses support those from previous studies and indicate that substitutions in Nsp12 are uncommon following treatment with remdesivir, and there is a strong barrier to high-level remdesivir resistance in patients with COVID-19 in the clinical setting. These data support the continued use of remdesivir for emerging variants of SARS-CoV-2 and in special patient populations.

## MATERIALS AND METHODS

### Clinical study

REDPINE was a Phase 3, randomized, double-blind, placebo-controlled, parallel-group multicenter study (ClinicalTrials.gov identifier NCT04745351; EudraCT registration no. 2020-005416-22), the details of which have been published elsewhere (16). Briefly, eligible participants were aged ≥12 years, weighed ≥40 kg, and had impaired kidney function (estimated glomerular filtration rate <30 mL/min/1.73 m^2^, including kidney failure requiring chronic dialysis, or ongoing AKI, defined as a 50% increase in serum creatinine within a 48-h period that was sustained for ≥6 h despite supportive care). Laboratory-confirmed SARS-CoV-2 infection was required, and participants must have been hospitalized with severe COVID-19, with symptom onset within 10 days prior to randomization. Exclusion criteria included requirements for invasive or noninvasive mechanical ventilation, extracorporeal membrane oxygenation, or dialysis for AKI.

The trial was conducted according to the International Council for Harmonisation (ICH) guideline for Good Clinical Practice and the principles of the Declaration of Helsinki. Protocols were approved by the relevant institutional review boards, and all participants provided written informed consent.

Participants were randomized (2:1) to receive intravenous remdesivir (200 mg on Day 1, followed by 100 mg/day on Days 2–5) or a matched saline placebo, both in addition to standard of care therapy (Fig. 1). Screening began on 13 March 2021, and the last participant visit was on 24 May 2022.

### Viral load analysis and mathematical modeling of viral load decline

The change from baseline in SARS-CoV-2 RNA viral load was compared in an *ad hoc* analysis between the remdesivir and placebo groups and between participants with and without SOT in the remdesivir group using an MMRM approach.

To model viral load decline, a statistical model of viral load was built to predict the effect of extending the treatment duration on viral load. To account for the hierarchical structure of the data and repeated measures within participants, a linear mixed-effects model was applied using the lme4 package in R (version 4.3.1).

Treatment model for participant *i* was specified as follows:


$$VL=I_{on}\left(\beta_{0}+\beta_{on}T+u_{oni}+\epsilon_{oni}\right)+(1-I_{on})(\beta_{i}+\beta_{post}∆T+u_{posti}+\epsilon_{post})$$

where VL is the viral load, *I_on_* is the indicator of participant on treatment (1 for participants on treatment; 0 for participants post-treatment), *β_0_* is the baseline viral load, *T* is the fixed-effect time on treatment, *ΔT* is the fixed-effect time post-treatment, *β_i_* is the viral load of the last on-treatment visit for participant *i*, *β_on_* and *β_post_* are the slopes of time for the on-treatment and posttreatment groups, respectively, *u_oni_* and *u_posti_* are the on-treatment and posttreatment mixed-effect terms for each participant *i* to account for variability within individuals, respectively, and *ϵ_oni_* and *ϵ_posti_* are random errors.

### Virologic resistance analyses

Nasopharyngeal swab samples were collected from participants until discharge at Days 1 (baseline), 3, 5, 7, 14, 21, and 29. SARS-CoV-2 viral load was determined using a validated RT-qPCR assay. Samples from participants (both remdesivir and placebo groups) with all-cause death or IMV, or with SARS-CoV-2 viral load at Day 14 that was higher than the LLOQ (2,228 copies/mL), were eligible for virologic resistance analyses. Participants whose samples did not have a SARS-CoV-2 viral load >LLOQ at Day 1 or ≥1 qualifying post-baseline sample were excluded from the virologic resistance analyses. SARS-CoV-2 lineage was determined using Pangolin software (v.4.1.1).

Full-genome next-generation deep sequencing of SARS-CoV-2 from the nasopharyngeal samples was conducted on Day 1, the last available day of treatment, and Day 14 or the last available visit with SARS-CoV-2 viral load >LLOQ using Illumina MiSeq or NextSeq platforms according to the ARCTIC protocol and Version 3 primer set (DDL Diagnostic Laboratory, the Netherlands). The primary genotypic analysis was the identification of emergent amino acid substitutions in the SARS-CoV-2 Nsp12 gene, with a secondary genotypic analysis of substitutions in SARS-CoV-2 Nsp8, Nsp10, Nsp13, and Nsp14 genes. Substitutions in Nsp12 identified from Baseline (Day 1) samples were compared with the SARS-CoV-2 reference sequence (WA1), and individual participant post-baseline amino acid substitutions were compared with their baseline sequences.

### Phenotypic analysis

Phenotypic analysis was performed using a replicon assay. All identified emergent amino acid substitutions in Nsp12 (i.e., Nsp12 substitutions not detected at baseline) were introduced into a SARS-CoV-2 replicon via site-directed mutagenesis and tested for susceptibility to remdesivir as compared with the SARS-CoV-2 SH01 (SARS-CoV-2/human/CHN/SH01/2020, GenBank MT121215) reference strain (35, 36). In nasopharyngeal swab samples where emergent amino acid substitutions in Nsp8, Nsp10, Nsp13, or Nsp14 were identified, phenotypic analysis was conducted using the replicon assay if there was also an Nsp12 amino acid substitution present or if requested by regulatory or health authorities.

Results of the phenotypic analysis were reported as fold change in the mean EC_50_ of the replicons relative to the baseline SARS-CoV-2 reference strain, with a cutoff for *in vitro* susceptibility of 2.5-fold change.

To calculate the replication capacity of the mutant replicons, the luciferase signal of the mutant replicon was divided by that of the wild-type replicon at 48 h post-infection.

The prevalence of SARS-CoV-2 substitutions was calculated based on the SARS-CoV-2 sequences available in the GISAID database as of 2 February 2026.

Any emergent Nsp12 amino acid substitutions were modeled on the cryo-electron microscopy structure of the SARS-CoV-2 replicase to determine the locations relative to the active site of Nsp12.

## Data Availability

Full sequence data are publicly available in the GenBank database (accession numbers PZ312170 to PZ312337). Gilead Sciences shares anonymized individual patient data upon request or as required by law or regulation with qualified external researchers based on submitted curriculum vitae and reflecting non-conflict of interest. The request proposal must also include a statistician. Approval of such requests is at Gilead Sciences’ discretion and is dependent on the nature of the request, the merit of the research proposed, the availability of the data, and the intended use of the data. Data requests should be sent to datasharing@gilead.com.

## SUPPLEMENTAL MATERIAL

Supplemental material aac.01912-25-s0001.pdf Tables S1 and S2; Fig. S1.

ASM does not own the copyrights to Supplemental Material that may be linked to, or accessed through, an article. The authors have granted ASM a non-exclusive, world-wide license to publish the Supplemental Material files. Please contact the corresponding author directly for reuse.
