# Supplementary material for: Virologic insights from the phase 3 REDPINE trial: remdesivir in renally impaired and immunocompromised patients with COVID-19
Source: Antimicrob Agents Chemother. 2026 Jul 10;70(8):e01912-25. doi: 10.1128/aac.01912-25 (PMC13436409; doi:10.1128/aac.01912-25)
Supplement: Supplementary file 1 [file aac.01912-25-s0001.pdf]

Supplementary Material

TABLE S1 LSM (95% CI) change from baseline[a] in SARS-CoV-2 viral load by study drug in participants with a history of SOT (Virology Analysis Set[b])

Study day	Remdesivir		Placebo		Treatment difference vs placebo	
	n	LSM (95% CI)[c]	n	LSM (95% CI)[c]	LSM (SE)[c]	Nominal *P* value
Day 1 (baseline)	29	–	5	–	–	–
Day 3	24	–0.61 (–1.38, 0.16)	3	–0.42 (–2.22, 1.37)	–0.19 (0.92)	0.840
Day 5	17	–1.01 (–1.82, –0.19)	3	–0.53 (–2.31, 1.24)	–0.47 (0.90)	0.603
Day 7	13	–1.16 (–2.04, –0.27)	3	0.32 (–1.45, 2.09)	–1.48 (0.96)	0.130
Day 14	11	–1.36 (–2.28, –0.45)	–	–	–	–
Day 21	5	–3.17 (–4.36, –1.97)	–	–	–	–
Day 29	4	–3.49 (–4.78, –2.20)	–	–	–	–

[a]LSM change from baseline in viral load and *P* values were determined using MMRM. LSMs are reported in $\log_{10}$ copies/mL.

[b]Virology analysis set included all participants who were randomized into the study, received ≥1 dose of the study drug, and had positive SARS-CoV-2 at baseline.

CI, confidence interval; LSM, least-squares mean; MMRM, mixed model for repeated measures; SARS-CoV-2, severe acute respiratory syndrome coronavirus 2; SE, standard error; SOT, solid organ transplantation.

TABLE S2 Remdesivir EC_{50} against SARS-CoV-2 reference and mutant Nsp13 replicons

**Nsp13 substitution**	**Mean (SD) remdesivir EC_{50}, nM**	**EC_{50} fold change from wild type[a]**
SH01 wild type	10.6 (4.0)	1.0
V6F	24.5 (2.4)	2.3
T127I	8.6 (0.1)	0.8
E136D	7.4 (0.8)	0.7
Y253H	5.3 (0.7)	0.5
S485L	8.6 (0.5)	0.8
Y541C	7.4 (2.6)	0.7

[a]The cutoff for *in vitro* susceptibility, which was based on the assay variability, was 2.5-fold change.

EC_{50}, half-maximal effective concentration; SARS-CoV-2, severe acute respiratory syndrome coronavirus 2; SD, standard deviation

FIG S1 EC_{50} curves of SARS-CoV-2 Nsp12 mutant replicons exposed to remdesivir[a]

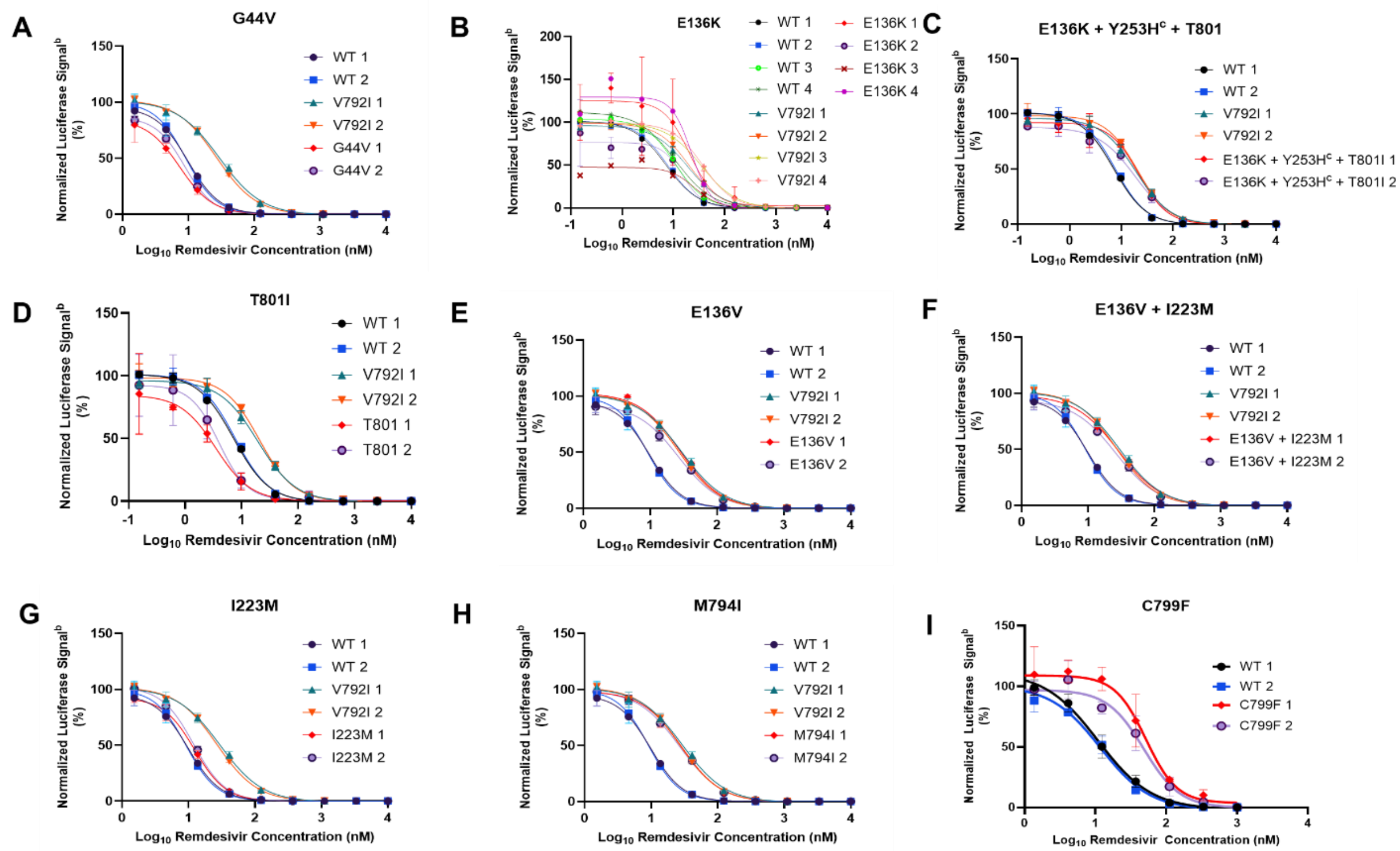

[a]Two experiments were performed for each replicon. The SARS-CoV-2 SH01 reference strain was included as a WT control. A replicon containing the V792I substitution was included as a positive control for all substitutions except C799F, which was tested before the establishment of the V792I positive control.

[b]Normalized to luciferase signal upon treatment with DMSO.

[c]Y253H was an emergent substitution in Nsp13.

DMSO, dimethyl sulfoxide; SARS-CoV-2, severe acute respiratory syndrome coronavirus 2.
